# Selected psychological aspects and medication adherence in oncological patients

**DOI:** 10.1002/cam4.2691

**Published:** 2019-12-14

**Authors:** Magdalena Gruszczyńska, Monika Bąk‐Sosnowska, Szymon Szemik

**Affiliations:** ^1^ Department of Psychology Chair of Social Sciences and Humanities School of Health Sciences in Katowice Medical University of Silesia in Katowice Katowice Poland; ^2^ Department of Nursing Propaedeutics School of Health Sciences Medical University of Silesia in Katowice Katowice Poland

**Keywords:** health locus of control, medication adherence, strategies of coping with stress

## Abstract

**Objective:**

The aim of this study was to analyze the relationships between selected psychological features and adherence to therapy in oncological patients.

**Methods:**

The study included 102 patients of oncological clinics, 66.67% of whom were female. The average (SD) age of the study subjects was 49.15 ± 18.16 years old. The following tools were used: Morisky Medication Adherence Questionnaire (MAQ‐4), Multidimensional Health Locus of Control, Coping Inventory for Stressful Situations, Personal Values Inventory (LWO), and a study‐specific survey questionnaire.

**Results:**

High MAQ‐4 scores were declared by 39.2% of the subjects, medium scores by 32.3% and low scores by 28.5%. The values were higher in female patients (*P* = .23), younger subjects (*P* < .001), and in individuals with higher education (*P* = .03). The greatest adherence was observed in subjects who placed their locus of control in chance (*P* = .022). Significant relationships were identified between the level of medication adherence and the use of avoidance strategies of coping with stress (*P* = .037), including the willingness to engage in social relationships (*P* = .04). It was demonstrated that the risk of noncompliance in the analyzed group was associated with a lower assessment of appearance (OR = 0.75) and health (OR = 0.78) on the scale of values.

**Conclusion:**

Medication adherence in oncological patients is related to the health locus of control, strategies of coping with stress, and the value assigned to appearance and health.

## Graphical abstract

Our study may be considered as a voice in the discussion on the medication adherence in oncological patients. Medication adherence is an important aspect in the treatment of oncological disorders, where the patient's cooperation with the medical team is crucial, not only for the success of the treatment but also for the quality of the patient's life. Medication adherence is determined by a number of factors, including those associated with the patient's psychological characteristics. Therefore, the purpose of this study was to analyze the relationships between selected psychological features and adherence to therapy, in oncological patients.

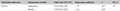

## 1 INTRODUCTION

Oncological diseases are the second most common causes of death globally. Based on the data from 2016, approximately 990 thousand people in Poland have cancer. The most common neoplasm in men is prostate cancer, whereas women are most frequently diagnosed with breast cancer, lung cancer, and colon cancer. The number of cancer incidents is slightly higher among males (82 250 vs 81 620 among females).^1^

Strict adherence to the prescribed therapy is one of the factors determining the effective treatment of any disease, including oncological disorders. If the therapy is conducted in a hospital setting, medication adherence appears to be a minor problem, as the responsibility for the administration of treatment is largely with the hospital personnel. When the treatment is carried out on an outpatient basis, without direct supervision of the medical personnel, medication adherence often becomes an issue adversely affecting the effectiveness of the therapy. Medication non‐adherence is a growing problem, which significantly contributes to treatment failure and exacerbation of disease symptoms.^2^, ^3^, ^4^, ^5^ The most common forms of non‐adherence include failure to collect prescriptions, resignation from treatment, delaying the start of the treatment, unintentional or intentional omission of individual doses, regular modification of dosing frequency, temporary use of a higher/lower number of doses, intervals in drug administration lasting a few days or longer, or premature discontinuation of therapy.^6^ Studies demonstrate that the phenomenon occurs irrespective of the type of disease, and is also observed in patients with neoplasms.^7^, ^8^, ^9^, ^10^ Regular use of oral medications in the prescribed doses is crucial in neoplastic disease. Depending on the type of neoplasm, a proper diet and change of lifestyle also often determine the success of the treatment. Despite the information provided by medical personnel and information campaigns, non‐adherence with these recommendations remains a growing trend.^11^, ^12^

The factors that affect medication adherence can be classified as patient‐related, healthcare system‐related, socio‐economic, therapy‐related, and disease‐related.^13^ Particularly important is the group of patient‐related factors, including demographics (female sex, older age and being married are associated with better medication adherence), patient's health‐related beliefs, understanding of the disease and its treatment, and psychological profile (cognitive skills, emotional status, and quality of social relationships).^14^, ^15^ Some studies demonstrate a relation between personality characteristics and therapy adherence.^16^, ^17^

The individual health locus of control also plays an important role in the decision‐making process. People with an external locus of control believe that what happens to them is due to chance or other people, whereas those with an internal locus of control believe in their own agency.^18^ This is reflected in their approach to health. Individuals with a high sense of responsibility for their health more often undertake pro‐health actions to prevent disease. However, in the context of mediation adherence, an inner locus of control is considered to be a negative factor. Patients who are convinced they have influence on their health are less willing to comply with the recommendations compared to people with an external locus of control.^19^, ^20^

As disease causes psychological stress, the patients’ individual styles of coping with stress also affect their co‐operation with the medical team. Task‐oriented individuals take actions to solve the problem. Emotion‐oriented patients in stressful situations concentrate on themselves and their emotional experience, demonstrating a propensity for fantasizing and wishful thinking. This reduces the emotional tension triggered by the stressful situation. The avoidance‐oriented style (AOS) is characterized by avoidance of thoughts, emotions, and experiences associated with a stressful situation. Two types of this style can be distinguished: engagement in substitute activity (eg, sleep, eating, computer games, watching TV, thinking about pleasant things), and seeking social relationships (SSR).^21^

As the above psychological factors are considered important for physical and mental health, few scientific reports exploring their relationships with medical adherence are available. The problem is particularly visible in the case of severe diseases, where the patient's co‐operation with the medical team is crucial, not only for the success of the treatment but also for the quality of the patient's life, or even survival. Therefore, the purpose of this study was to analyze the relationships between selected psychological features and adherence to therapy, in oncological patients.

## 2 METHODS

### 2.1 Participants

The study involved patients from oncological clinics in the Silesian region of Poland. The inclusion criteria were as follows: (a) age over 18 years; (b) diagnosis of a neoplastic disease received at least 1 month before; (c) being a patient of an oncological clinic; (d) medical prescription for regular medication; and (e) Provision of an informed consent to participate in the study. The exclusion criteria included: (a) intellectual disability; (b) mental disease or a history of mental disease; (c) first visit to an oncological clinic; and (d) withdrawal of the informed consent to participate in the study.

A total of 120 subjects were enrolled in the study initially; 9 patients refused to participate, and 11 patients only partially completed the questionnaires. The final analysis involved 102 patients.

### 2.2 Measures

The study was conducted using diagnostic survey questionnaires. The following standardized questionnaires were used to assess psychological characteristics:

*Morisky Medication Adherence Questionnaire* by Morisky, Green, Levine: this scale assesses the degree to which patients adhere to a physician's recommendations regarding medication. It consists of four items, to which patients can respond “yes” or “no.” The total score allows the patient's adherence with therapy to be determined, as low (0‐1 points), moderate (2‐3 points), or high (4 points). The cut points were proposed by the author of the scale^22^
*Multidimensional Health Locus of Control Scale* by Wallston, Wallston, DeVellis, in the Polish version by Juczynski; it consists of 18 items, assessed on a 6‐point scale, regarding general expectations in three dimensions of the locus of control: internality, powerful others externality, and chance externality. Higher scores on each subscale indicate a stronger belief that the factor affects health status.^23^
*Coping Inventory for Stressful Situations *by Endler and Parker, in the Polish adaptation by Szczepaniak, Wrześniewski and Strelau; the questionnaire consists of 48 statements regarding various behaviors that can be demonstrated by people in stressful situations. The patient assesses on a 5‐point scale the frequency of engaging in a given action in difficult, stressful situations. The scores are presented on three scales: task‐oriented style, emotion‐oriented style, and AOS. The last one is observed in two forms: engagement in substitute activities or SSR.^24^
*Personal Values Inventory (LWO)* **,** developed by Juczyński, is a questionnaire comprising 10 categories of personal values: love, good health, sense of humor, intelligence, knowledge, joy, courage, goodness, attractive appearance, and wealth. The patient is asked to rank the values from the most important (score 1) to the least important (score 10).^23^

The questionnaire developed by the authors for the purpose of this study comprised 13 closed questions regarding anthropometric measures (body weight, height, waist circumference), socio‐demographic data (sex, age, place of residence, education, occupational status, family status, religiousness), course of the disease (time since the diagnosis, duration of the treatment, recommendations related to medication and other therapeutic recommendations), and lifestyle (tobacco smoking, alcohol consumption) of a neoplasm was longer than 24 months. Also the duration of treatment was usually over 2 months.

### 2.3 Study organization

The study was conducted in hospital oncological clinics in the Silesia region (Poland). All patients who attended the clinics in the period of November 5‐9, 2018 were invited to participate in the survey. The patients were informed about the aim of the study, its anonymous and voluntary character, and lack of payment for participation. Subjects received the questionnaires in a paper form. They completed them personally, without any time limit, in the clinic, while waiting to be seen. The final analysis involved 102 patients. The study was conducted as part of the statutory work in Medical University of Silesia in Katowice (KNW‐1‐033/N/8/2); the research project received approval from the competent Bioethics Commission (approval no. KNW/0022/KB/170/17).

### 2.4 Statistical analysis

The analysis involved the descriptive and analytical methods available in the Statistica 13.3 software. Central trend measurements and measures of dispersion were used for quantitative variables. The Shapiro‐Wilk test was used to assess normality of distribution of the variables identifying psychological characteristics. To interpret quantitative variables in independent groups defined by selected factors, non‐parametric tests were used, due to the deviations in the distribution of variables, compared to a normal distribution (Mann‐Whitney *U* test and Kruskal‐Wallis ANOVA). In addition, the statistically significant correlations identified by the Kruskal‐Wallis ANOVA test were verified using intergroup multiple comparisons. Distributions of qualitative variables were compared using the chi‐square test and contingent tables. The statistical significance level in all analyses was set at *P* < .05.

The results of the simple analyses were verified by logistic regression. Regression model was developed using backward elimination of the statistically insignificant predictors. The variables that identified the psychological characteristics of respondents were entered into the model. The Hosmer‐Leme show test was used to assess the model's quality, where *P* > .05 indicated a good fit between the model and the data.

Selected categories of variables were transformed into dichotomous values in order to facilitate statistical interpretation and regression analysis.

## 3 RESULTS

The majority of the study group were women and patients who were married or in a partnership. Regarding the social status and living conditions, the majority of respondents were unemployed or professionally inactive, and had children. The average age of the study subjects was 49.15 ± 18.16 years old. Table 1 presents selected descriptive statistics regarding the basic demographics of the study subjects.

**Table 1:** Selected descriptive statistics for the study group (N = 102)

Variable	N	%
Sex		
Female	68	66.7
Male	34	33.3
Marital status		
In a relationship	65	65.7
Single	37	36.8
Education		
Higher	32	31.4
Secondary school	43	42.1
Below‐secondary school	27	26.5
Residence		
Rural area	17	16.7
Town with a population of up to 50 thousand	33	32.4
Town with a population of 51 thousand to 200 thousand	24	23.5
Town with a population of over 200 thousand	28	27.45
Employment		
Employed	38	37.3
Unemployed or professionally inactive	64	62.7
Having children		
Yes	69	67.6
No	33	32.4

Mean BMI in the study group was 26.50 ± 5.34. Over half of the subjects (50.80%) declared that the time since the diagnosis of a neoplasm was longer than 24 months. The most common type of cancer in study group was breast cancer, colon cancer, lymphoma, and myeloma. The duration of the treatment was usually over 2 months (47.06%).

The largest group of patients (39.2%) declared a high level of medication adherence, whereas moderate and low adherence levels were declared by similar rates of subjects (moderate—32.3% and low—28.5%).

The degree of medication adherence was statistically significantly varied according to the sex, age, and education of the subjects (Table 2). High medication adherence was observed more often in females than in males (82.5% vs 17.5%), in patients up to 53 years old compared to older subjects (77.5% vs 22.5%), and in respondents with higher education compared to those with a lower education level (47.5% vs 12.5%). No significant differences regarding medication adherence were found in the study group between patients in terms of marital status, place of residence, occupations status and with or without children.

**Table 2:** Statistical significance in a chi‐square test for correlation between degree of medication adherence and demographic variables (N = 102)

Variable	Degree of medication adherence (low vs moderate vs high)
Sex (female vs male)	0.230
Age (18‐49 vs 50‐77)	**<0.001**
Marital status (in a relationship vs single)	0.817
Education (higher vs secondary school vs occupational)	**0.030**
Place of residence (rural area vs town of up to 50 thousand vs town of 51‐200 thousand vs town of over 200 thousand)	0.938
Occupational status (employed vs unemployed or professionally inactive)	0.295
Having children (yes vs no)	0.204

Statistically significant values are in bold.

Table 3 presents data regarding the mean scores for the studied psychological variables. In addition, the variation is presented in the scores obtained for individual variables according to the degree of medication adherence during treatment. The analysis revealed significant differences in the degree of adherence in the group of patients who believed that chance affects their health the most (*P* = .022). The stronger their belief in the influence of chance, the more they adhered to the physician's recommendations. In addition, significant relationships were identified between the level of medication adherence and the use of avoidance strategies (*P* = .037), including the willingness to engage in social relationships in stressful situations (*P* = .040). The most compliant patients demonstrated the highest level of avoidance and most frequently chose social relationships as a way to avoid confrontation with the stressor.

**Table 3:** Scores for individual psychological variables according to medication adherence during treatment (N = 102)

Variable	Whole group	Degree of medication adherence		
		Low (Mean ± IQR)	Moderate (Mean ± IQR)	High (Mean ± IQR)
Personal values				
Love	8.00 ± 5.50	9.00 ± 4.00	8.00 ± 5.00	8.00 ± 4.50
		*P* = .579		
Health	9.00 ± 5.00	9.00 ± 6.00	10.00 ± 3.00	9.00 ± 4.50
		*P* = .466		
Sense of humour	5.00 ± 3.00	5.00 ± 4.00	5.00 ± 5.00	5.00 ± 3.00
		*P* = .991		
Intelligence	6.00 ± 4.00	6.00 ± 3.00	6.00 ± 5.00	6.00 ± 3.00
		*P* = .703		
Knowledge	5.50 ± 3.00	6.00 ± 2.00	5.00 ± 3.00	5.50 ± 3.00
		*P* = .774		
Joy	6.00 ± 3.00	6.00 ± 3.00	6.00 ± 3.00	6.00 ± 4.00
		*P* = .863		
Courage	4.50 ± 4.00	5.00 ± 5.00	5.00 ± 4.00	4.00 ± 5.00
		*P* = .440		
Goodness	6.00 ± 4.00	5.00 ± 3.00	7.00 ± 3.00	5.00 ± 5.00
		*P* = .194		
Appearance	2.50 ± 5.00	2.00 ± 4.00	4.00 ± 3.00	3.00 ± 5.50
		*P* = .244		
Wealth	2.00 ± 5.00	2.00 ± 5.00	2.00 ± 3.00	2.00 ± 5.50
		*P* = .946		
Health control				
Inner‐control	19.00 ± 9.00	18.00 ± 6.00	19.00 ± 9.00	19.50 ± 9.50
		*P* = .962		
Influence of others	19.00 ± 6.00	17.00 ± 6.00	17.00 ± 7.00	20.00 ± 6.50
		*P* = .130		
Chance	20.00 ± 9.00	20.00 ± 8.00	19.00 ± 6.00	23.00 ± 9.00
		***P* = .022**		
Style of coping with stress				
Task‐oriented	56.50 ± 10.00	56.00 ± 13.00	56.00 ± 11.00	57.00 ± 9.00
		*P* = .225		
Emotion‐oriented	44.00 ± 14.00	44.00 ± 16.00	43.00 ± 12.00	44.50 ± 13.00
		*P* = .602		
Substitute activities	20.00 ± 8.00	21.00 ± 4.00	18.00 ± 8.00	21.00 ± 9.00
		*P* = .109		
Social relationships	16.00 ± 6.00	16.00 ± 5.00	15.00 ± 5.00	16.50 ± 5.50
		***P* = .040**		
Avoidance‐oriented	36.00 ± 12.00	37.00 ± 11.00	33.00 ± 11.00	37.50 ± 12.00
		***P* = .037**		

Note

*P*—statistical significance for Kruskal‐Wallis ANOVA test. Statistically significant values are in bold.

Finally, the results of a simple analysis of the relationships between medication adherence and individual psychological characteristics of the study subjects were verified by logistic regression. Predictors of non‐adherence for oncological patients were sought. The results are presented in Table 4. It has been demonstrated that non‐adherence in the analyzed group was associated with a lower assessment of appearance (OR = 0.75) and health (OR = 0.78) on the scale of values.

**Table 4:** Results of the analysis multiple variables for the correlation between medication adherence during oncological treatment and individual independent variables (N = 102)

Medication adherence	Independent variable	Odds ratio (95% PU)	Regression coefficient	*P*	H‐L *P*
No/Yes	Appearance	0.75 (0.60, 0.95)	−.27	.017	.35
	Health	0.78 (0.63, 0.95)	−.24	.018	

Abbreviations: H‐L, Hosmer‐Leme show test (*P* > .05 indicates a good fit between data and model), *P*, statistical significance.

## 4 DISCUSSION

Good collaboration between the patient and the physician during the treatment process is paramount for successful therapy. Strict adherence to the physician's recommendations is among the most important factors in the treatment of oncological diseases. Studies regarding the degree of medication adherence over the past few years have demonstrated that for many patients the systematic use of medications and the administration of the prescribed forms of therapy pose significant challenges. The problem occurs irrespective of the type of disease or availability of healthcare.^5^, ^7^, ^10^

Our findings revealed that the higher degrees of medication adherence in the analyzed group were associated with sex, age, and education level. This is consistent with the results of a literature review from the years 2000‐2009 conducted by Kardas et al, which demonstrated a higher level of medication adherence in females and patients with higher education.^14^ Moreover this result can be explained by the relationship between the variables and health‐related behaviors. Medication adherence is an example of a pro‐health behavior, and the majority of studies demonstrate that females and people with higher education take better care of their health.^14^

Among the analyzed factors potentially affecting medication non‐adherence a high assessment of appearance, distinguished from the set values appreciated in life, was found to be significant. This observation may be surprising, considering that the competing values included health or love. Oncological disease and its treatment process is usually associated with changes in a patient's appearance. Hair loss, skin pallor, emaciation or, on the contrary, drug‐induced swelling, are only some of the factors that may affect the patient's perception of their body.^25^ In her studies involving 30 patients of hematology departments following chemotherapy, Tomasiewicz revealed that their body image was worse than in the group of healthy women, with similar values assigned to appearance in both groups. The patients largely followed the recommendations regarding personal hygiene in order to improve their appearance and accelerate the treatment process.^26^ It appears that the eagerness to maintain or return to the looks from the time before the disease may contribute to medication adherence. This finding should be important for the personnel taking care of oncological patients, and who have to deal with the side effects of the disease and its treatment. Considering how important appearance is for patients, all efforts should be made to reduce the visible effects of the therapy, as this can increase its effectiveness and motivate patients to receive regular treatment.

Another factor analyzed by the authors was health locus of control. In psychological theory, an external health locus of control is the most important factor contributing to medication adherence, followed by the belief that chance is responsible for our health status. Patients with inner locus of control, who are convinced they have influence on their health, are less willing to comply with recommendations compared to people with an external locus of control.^19^, ^20^ Our findings demonstrated that a high degree of medication adherence was associated with the belief that health status is determined by chance. This is consistent with the results shown by Kurowska and Kalawska in a study involving 98 patients following a mastectomy, in which chance had the highest score among all dimensions of the health locus of control.^27^ Similar outcomes were presented by Milaniak, who studied 50 oncological patients using an identical MHCL test. An external health locus of control was demonstrated in the highest number of subjects (27.6 ± 5.1 points), followed by the view that health status is determined by chance (25.0 ± 7.2 points).^28^

The authors of the present study did not include duration of treatment in the analysis of results regarding the health locus of control. Patients receiving long‐term treatment may have a tendency to shift responsibility away for the therapeutic process. After a number of therapies, they might demonstrate fatigue and discouragement, and the belief in chance as the source of success may play the role of a defense mechanism. This would allow them to free themselves and the physician from the burden of responsibility for a potential treatment failure. From the point of view of an oncological patient, the scrupulous and meticulous adherence to the therapy could co‐occur with the belief that fate or chance would be favorable and hence ensure recovery. However, this hypothesis requires further analysis.

When dealing with a neoplastic disease, the style of coping with stress, activated in difficult and critical situations, is of great importance. The diagnosis of a neoplastic disorder itself may evoke the impressions of pain, suffering and death, regardless of the further course of the disease, as the patient's behavior and attitude to therapy is largely determined by the way they prepare for the situation. Dealing with stress is a response to a given situation, and is highly individualized.^24^, ^29^ The style of coping with stress means “a relatively permanent tendency in different situations to use the coping strategies specific for an individual, in order to eliminate or reduce stress.”^29^

In the studied group of oncological patients, the subjects demonstrating high medication adherence revealed the strongest reliance on avoidance strategies, in particular on engagement in social relationships. This observation suggests that patients have a tendency to avoid of thinking about the stress off illness. This is in line with the theory of Endler and Parker where the AOS is manifested, among others, in SSR and support.^24^

The analysis of the literature from the years 2000‐2009 conducted by Kardas et al confirmed that social support contributes to medication adherence.^14^ Engagement in social relationships often satisfies the need for closeness, and allows one to move beyond the stressful reality related to the treatment.

Considering the results of previous analyses regarding the health locus of control, a close relationship between the analyzed factors is observed. Individuals who adhere to the therapy to a high degree tend to perceive chance as the determinant of therapeutic success and health status. They also demonstrated the AOS of coping with stress. In leaving their health to chance, patients avoid the responsibility for the results, as well as confrontations with difficult situations and with their emotions associated with the entire treatment process. They follow the physician's recommendations, at the same time making the effort not to confront the emotional or remedial aspect of the situation, and avoid getting their hopes high—probably taking into account that, due to the nature of the disease, recovery may not be possible. The results of our study indicating the co‐occurrence of medication adherence, the AOS of coping with stress and considering chance to be the health locus of control requires further study involving a larger patient group as well as other disease units. It may appear that the observed correlation is specific for oncological patients, and is not found in other diseases.

### 4.1 Study limitations

All psychological variables analyzed in the study are of a personality nature, which means that they remain relatively constant and do not change under the situation. In contrast, medical adherence may be influenced by situational conditions and factors as: the type of disease, its duration, the stage of illness and prognosis, the treatment method used, the level of acceptance of own illness, or the complexity and nuisance of individual recommendations. Interpersonal factors such as the quantity and quality of social support received by the patient, and even the effectiveness of his communication with the attending physician are also important. None of the mentioned factors was included in the conducted study. Emphasis was placed not on the conditions of medication adherence but on the personal factor, what, however, does not take into account the full picture of the phenomenon under investigation. Moreover the data on the type of cancer, type of treatment, and the type of medication have been not analyzed in our study. Therefore, the present results should be viewed as having a pilot nature, illustrating only a portion of reality and providing a starting point for further, more extensive analyses.

### 4.2 Clinical implications

Medical personnel and family members caring for patients with oncological diseases should take into consideration that:

1. Patients should be included in the treatment decision‐making process only to the extent desired by them; if patients have an external locus of health control, their willingness to cooperate with the therapeutic team will be limited;
2. Patients should not be obligatorily confronted with details regarding their health and prognosis, if they did not clearly ask of it, because in people with a unique style of coping with stress, the information intensifies, rather than reducing the level of anxiety;
3. Patients should have the possibility to maintain social relations, also during hospitalization, because social support has a positive effect on health and well‐being.

Doctors deciding about the method of treating a patient with oncological disease should not ignore the importance of the external appearance for the patient's well‐being and his quality of life. Whenever possible, the extent of injury to the patient as a result of therapy should be minimized, and if it happens, the opportunity to reconstruct of lost body aspect (eg, amputated breast) should be provided. The method of treatment that allows the patient to maintain a satisfactory appearance increases the chance of his cooperation with medical staff in the area of adherence to recommendations.

## 5 CONCLUSIONS

Medication adherence in oncological patients is related to:

1. Placing the health locus of control in chance.
2. AOSs of coping with stress, in particular the engagement in social relationships.
3. High rating assigned to appearance and health in the hierarchy of personal values.

## CONFLICT OF INTEREST

All authors declare that they have no conflicts of interest.

## Data Availability

The data that support the findings of this study are available on request from the corresponding author. The data are not publicly available due to privacy or ethical restrictions.
